# Dare to Compare. Development of Atherosclerotic Lesions in Human, Mouse, and Zebrafish

**DOI:** 10.3389/fcvm.2020.00109

**Published:** 2020-06-30

**Authors:** Viviana L. Vedder, Zouhair Aherrahrou, Jeanette Erdmann

**Affiliations:** ^1^Institute for Cardiogenetics, University of Lübeck, Lübeck, Germany; ^2^DZHK (German Centre for Cardiovascular Research), Partner Site Hamburg/Kiel/Lübeck, Lübeck, Germany; ^3^University Heart Centre Lübeck, Lübeck, Germany

**Keywords:** atherosclerosis, animal models, mouse, zebrafish, immune response, human disease, APOE, LDLR

## Abstract

Cardiovascular diseases, such as atherosclerosis, are the leading cause of death worldwide. Although mice are currently the most commonly used model for atherosclerosis, zebrafish are emerging as an alternative, especially for inflammatory and lipid metabolism studies. Here, we review the history of *in vivo* atherosclerosis models and highlight the potential for future studies on inflammatory responses in lipid deposits in zebrafish, based on known immune reactions in humans and mice, in anticipation of new zebrafish models with more advanced atherosclerotic plaques.

## Introduction

Cardiovascular diseases (CVDs) remain the leading cause of death worldwide (1). Research on atherosclerosis is of great clinical importance, because it increases the risk of CVDs.

**Figure 1:**
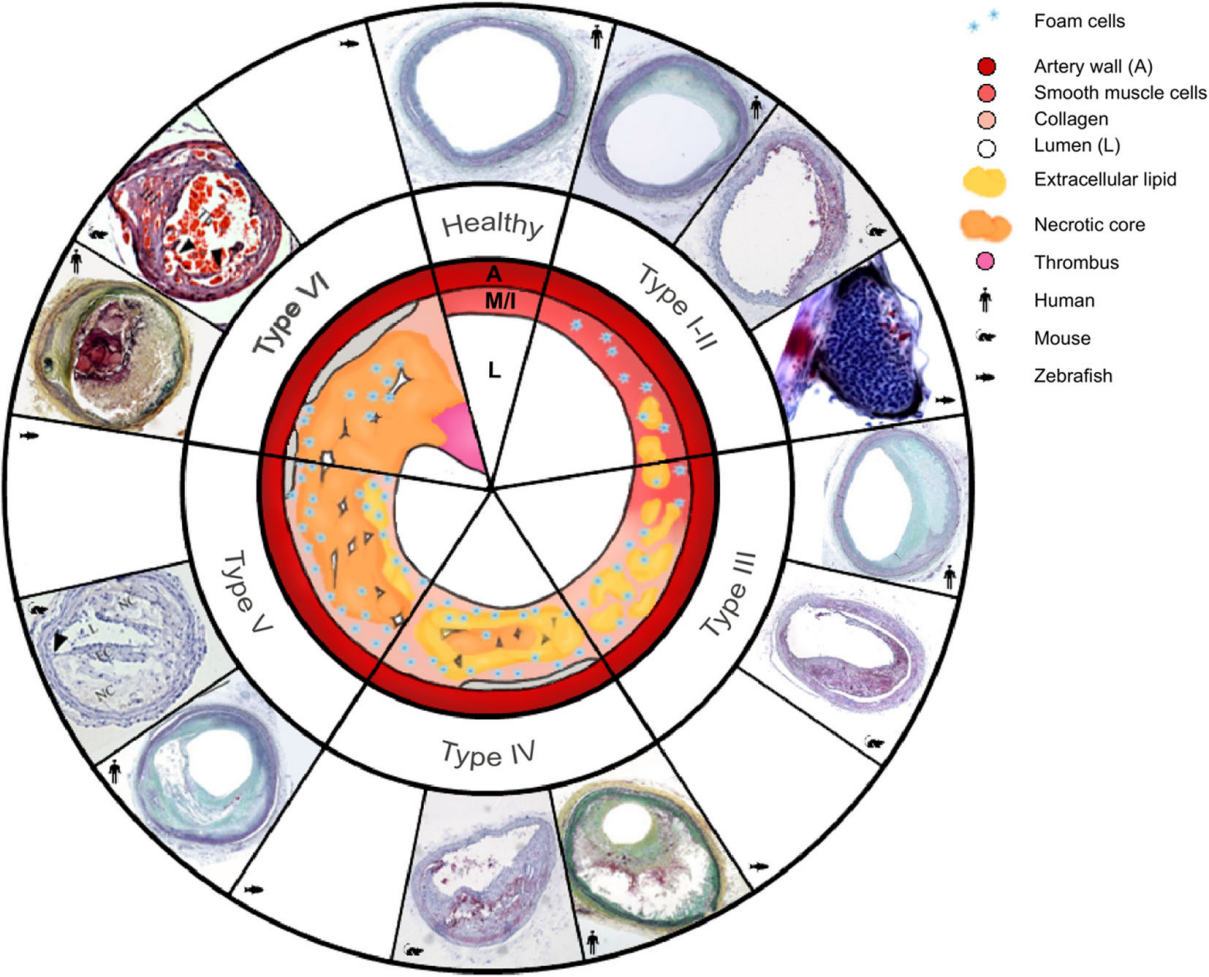
Morphology of the six types of atherosclerosis in human, mouse, and zebrafish: a schematic overview with a comparison of micrographs. Healthy arteries have an adventitia surrounding the media (M), a very thin intima (I) layer, and a large lumen (L). As atherosclerosis initiates and progresses, different types are classified as follows: Type I, intima thickening; type II, fatty streaks, with or without macrophages; type III, intermediate lesion; type IV, advanced atheroma; type V, fibroatheroma; type VI, complicated plaques with surface defects, leading to plaque rupture. The type VI mouse lesion image shows an unstable plaque in the right common carotid artery in the tandem stenosis mouse model, and it is used to represent future mouse plaque rupture models. Micrographs representing human atherosclerosis, are from Yahagi et al. (2); micrographs of *ApoE*^−/−^ mouse type I–IV atherosclerosis in the brachiocephalic artery and zebrafish belong to the Institute for Cardiogenetics. A set of micrographs of mouse types V and VI were taken from Chen et al. (3) Permission was granted for the use of all micropgraphs.

Atherosclerosis is a chronic inflammatory disease that leads to myocardial infarction and cerebrovascular events. In addition to independent risk factors, such as blood pressure, hypertriglyceridemia, diet, and smoking, genetic disorders can have major pro-atherosclerotic effects. Atherosclerotic plaques develop when low density lipoproteins (LDL) accumulate in the sub-endothelial space of the arterial wall, where they are oxidized, leading to inflammatory responses (Figure 1). Then, dendritic cells, T-cells, and monocyte-derived macrophages are recruited to the affected area. Macrophages absorb oxidized LDL (oxLDL), which can accumulate and convert macrophages into foam cells, which in turn stimulate the proliferation and migration of vascular smooth muscle cells (VSMCs) into the developing plaque. As the plaque grows, its stability may change. Decreasing stability potentially leads to plaque rupture, which triggers a thrombosis cascade. The consequences of rupture include occluded arteries, ischemic events, myocardial infarction, stroke, and sudden death. Several animal models have been developed to further understanding of the pathomechanisms underlying CVD, especially atherosclerosis. The ideal disease model should develop various stages of atherosclerotic plaques and have human-like lipid metabolism and immune responses. In this review, we discuss rabbit, rat, mouse, and zebrafish, four of the most commonly used animal models for atherosclerosis. Primarily, we compared mice and zebrafish as models for human patients.

## Atherosclerosis in Human, Mouse, and Zebrafish

### Pathogenesis of Atherosclerosis in Humans

Atherosclerosis pathogenesis comprises a complex interaction of hemodynamics, lipid metabolism, immune response and vascular injury response. Human atherosclerosis was classified into six fluent developmental stages, Type I-VI [Figure 1; (4)]. Lesions usually develop in regions of the arterial tree with local flow field disturbances leading to low shear stress, that induces endothelial cell (EC) activation and thereby the development of type I atherosclerosis (intimal thickening) (5, 6). Additionally, flow field changes contribute to accumulation of excess LDL in the sub-endothelial space of the arterial wall (LDL concentration polarization), where LDL is oxidized to oxLDL (5, 7). This reaction induces a T-helper type 1 (T_H_1)-driven inflammatory response, which stimulates expression of adhesion proteins, such as vascular cell adhesion molecule (VCAM)-1, E-selectin, P-selectin, and chemokines, including CCL2, CCL5, and CX3CL1 (8). One of the key players in plaque development is interferon-γ of T_H_1, which elevates chemokine secretion and upregulates adhesion molecule levels. Subsequently, dendritic cells, T-cells, and monocytes are recruited to the affected area in the arterial wall. Endothelial cells of normal and atherosclerotic arteries, as well as monocyte-derived macrophages, express various pattern recognition receptors (PRRs), including toll-like receptor (TLR) 1, TLR2, TLR3, TLR4, TLR5, TLR7, and TLR9 and inflammasomes [e.g., nucleotide oligomerization domain-, leucine-rich repeat-, and pyrin domain-containing protein-3 (NLRP3)] (8, 9). The latter are damage-associated molecular pattern (DAMP)-activated intracellular innate immune signaling complexes, that activate pro-inflammatory transcription factors such as NF-κB and p53 (6, 10). Dendritic cells in healthy arterial walls silence T-cells, whereas in plaques, activation of danger signals, such as DAMPs [e.g., oxLDL, necrotic cell debris, cholesterol crystals; (11, 12)], promotes dendritic cell tolerance to T-cell antigens. Concurrently, monocytes differentiate into macrophages that absorb oxLDL through their scavenger receptors (SRs), such as CD36, MARCO, SRA-1 and -2, and SR-B1. SRs mediate the import of oxLDL via fluid phase uptake, CD36-dependent endocytosis, or micropinocytosis (13). Atherogenesis can be mediated by cholesterol crystals that directly and indirectly prime and activate macrophages via neutrophil extracellular traps (NETs) and NLRP3s (6, 14). When oxLDL accumulates as cytosolic droplets, it converts macrophages into foam cells (15). Newest *in vitro* findings, that are still under discussion, indicate that oxLDL activates NLRP3 inflammasomes and restricts autophagy, thereby reducing the inflammatory response. One explanation is, that low level oxLDL stimulation dampens the inflammatory response, whereas hyperlipidemia ultimately leads to chronic inflammation (12, 16). The transition from type I to type II atherosclerosis, the fatty streak, is very fluid; accordingly, we chose to merge these types together in Figure 1.

In type III atherosclerosis (intermediate lesions), extracellular lipid droplets are scattered throughout the intima. Foam cells present antigens [e.g., heat-shock protein 60 (Hsp60), interleukin-6 (IL-6) and IL-1ß] to immune cells, such as monocytes and T-cells, thereby stimulating the proliferation of VSMCs in the developing plaque (17). Eventually, extracellular lipids concentrate into a growing lipid core (type IV). Concurrently, apoptotic foam cell membranes stimulate endothelial cells to recruit additional monocytes, creating an inflammatory positive-feedback loop that leads to the formation of a necrotic core (type V) (6, 14). Additionally, migrating VSMCs contribute to the development of a fibrous cap. Lesion growth eventually restricts blood circulation and thereby increases blood pressure, which in turn can lead to hypertension and thrombus formation.

In type VI atherosclerosis, the complicated plaque, lesions grow further until the artery is sealed and blood flow is prevented, resulting in myocardial infarction. Low shear stress is not only an induction, but also a progression factor of atherogenesis, that reduces collagen fibers, increases the necrotic core and causes thinning of the fibrous cap. Taken together, this makes the fibrous cap more susceptible to tensile stress and can lead to rupture, which triggers a thrombosis cascade that occludes the artery and causes ischemic events, myocardial infarction, unstable angina, stroke, acute coronary syndrome, and sudden death (18).

### Overview of Animal Models Over the Last 100 Years

**Figure 2:**
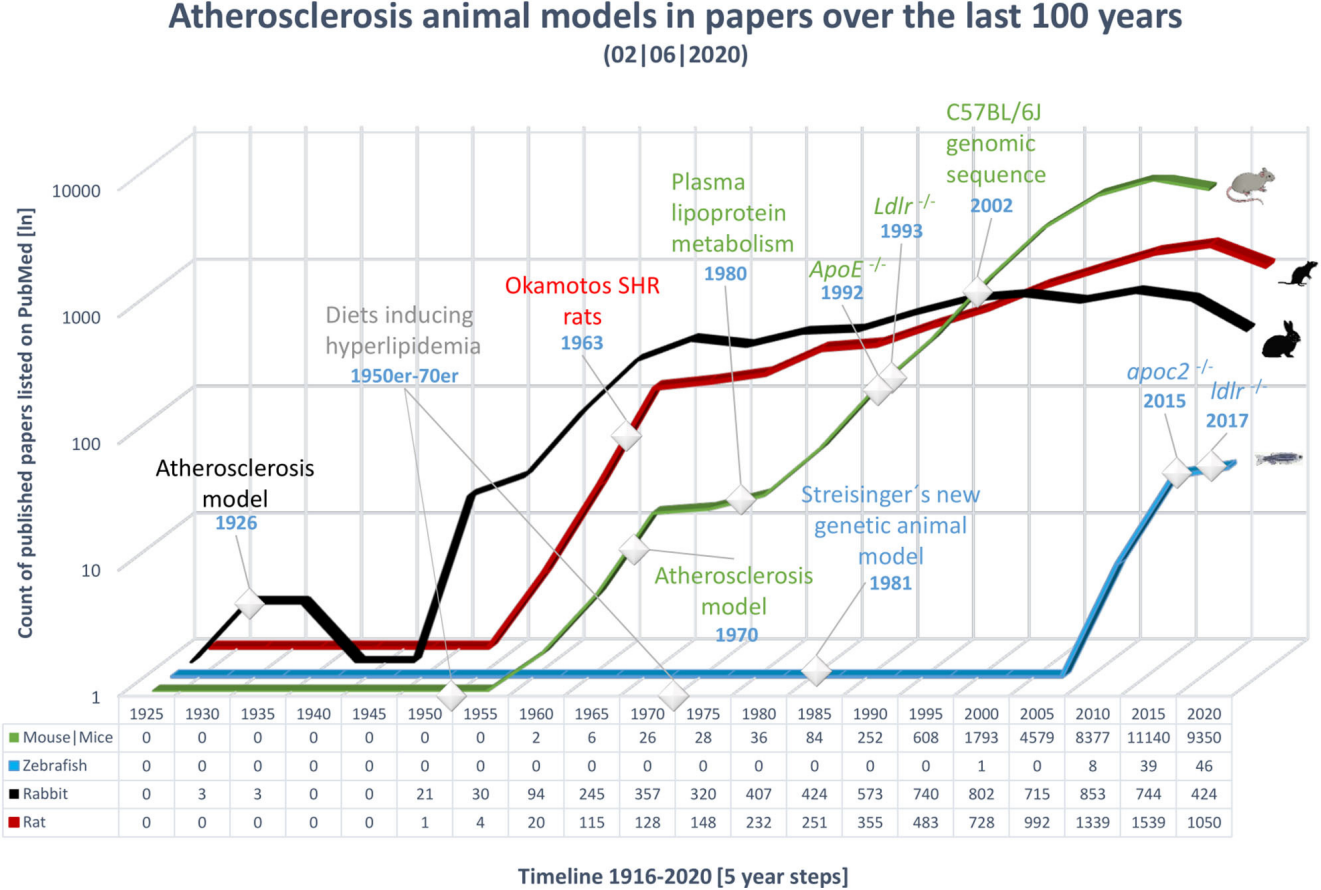
Overview of publications over the last 100 years on the topic of atherosclerosis in various animal models. The x-axis shows time, from 1921 to 2018, in 5 year bins; the last time point includes only 3 years. The most important events in the history of atherosclerosis research have been marked. The y-axis shows the number of publications in PubMed, on a logarithmic scale; an exact count is shown below the timeline for each animal model. Results were gathered using the MeSH term atherosclerosis in combination with the model to include a wide range of publications. Black, rabbit; red, rat; green, mouse; blue, zebrafish.

Over the last 100 years, many processes involved in the pathogenesis of atherosclerosis have been revealed; however, many aspects of this disease still require clarification. In 1908, Ignatowski discovered the potential of rabbits as an atherosclerosis model by describing the thickening of the intima accompanied by the formation of large cells in the aorta of rabbits fed an animal protein-enriched diet (19–21). In 1926, Clarkson and Newburgh were the first to publish on atherosclerosis using rabbits. They evaluated the effect of different diets varying in cholesterol and protein concentration and discovered that high-cholesterol diet (HCD) as well as high protein diet led to atherosclerosis and hypercholesterolemia (22). Further research on diet-induced modifications of arteries was performed from 1926 to 1935. After World War II ended in 1945, new animal models for CVD emerged; first the rat, later the mouse, and in the last 20 years, the zebrafish (Figure 2).

From the 1950s to 1970s, various diets capable of inducing hyperlipidemia were developed and tested in rats and rabbits (21). Two examples of prominent diets used to experimentally induce atherosclerosis include the Paigen diet (PD) (15% fat, 1.25% cholesterol, and 0.5% cholic acid) and the Western-type diet (WTD) (21% fat by weight, 0.15% cholesterol, and no cholic acid) (21). Hence, investigations of diet-inducible atherosclerosis have made critical contributions to the understanding of the pathogenesis of this condition.

In the 1970s and 1980s, intensive investigations of atherosclerosis began in mice. The characterization of plasma lipoprotein metabolism in the 1980s, in combination with the emergence of transgenic technologies in the 1990s, led to the development of the transgenic knockout mouse lines: *ApoE*^−/−^ in 1992 (23) and *Ldlr*^−/−^ in 1993 (24). Further, the complete DNA sequence of the diet-sensitive mouse strain, C57BL/6, was published in 2002 (25).

In 1981, Streisinger et al. were the first to show that forward genetics can be performed in zebrafish (26). The first zebrafish study to mention atherosclerosis, in the context of loss of the C-terminal end of the mammalian lipoprotein lipase protein, was published in 1996 (27). Starting in 2007, the number of studies published has increased, including research that led to the first genetic model of hyperlipidemia, the *apoc2*^−/−^ zebrafish mutant (28). Recently, Liu et al. reported the first *Ldlr*^−/−^ zebrafish mutant, a genetic model of hypercholesterolemia (29). Lipid metabolism and lipid trafficking genes are conserved in zebrafish, making this mutant an important tool for atherosclerosis studies (30).

We observed that the focus on animal models for CVDs has shifted over the last 100 years: from rabbit and rat, to mouse, and, possibly soon, to zebrafish. As mice and zebrafish need less space than rabbits and rats, their husbandry is cheaper. Furthermore, they are easier to genetically manipulate. Therefore, in the following sections we critically evaluate the mouse and zebrafish as the most prominent animal models for atherosclerosis.

### Mouse

Due to its low associated costs, easy maintenance, and breeding, extensive tools for genetic manipulation, and the ability to control its diet to rapidly induce atherosclerosis in various mutants, the mouse (*Mus musculus*) is a very useful model. Physiological similarities based on its phylogenetic relationship to humans, in addition to other factors mentioned below, make the mouse an important model for scientific experiments and the development of therapies (31). Therefore, we described the advantage and disadvantage of using genetically modified models in research and drug development.

Wild-type (WT) rodents are mostly resistant to atherosclerosis when fed a HCD (15); however, the development of inbred strains, such as C57BL/6J, C57L/J, and DBA/2J (32), has enabled atherosclerosis research in mice. Forward and reverse genetics play crucial roles in the development of atherosclerosis models. Knockouts, knockins, knockdowns, and transgenic modifications can be generated by various methods, including the CRISPR/Cas9 system, the Cre-loxP-system, Vivo-Morpholinos (MOs), lentiviral infection, and ethylnitrosourea (ENU) mutagenesis (33, 34). Genetic manipulation yielded the two most commonly used mouse models of atherosclerosis, namely *Ldlr*^−/−^ and *ApoE*^−/−^ (Table 1).

**Table 1:** Phenotypes and traits of mouse and zebrafish atherosclerosis models.

**Model**	**Induction (age)**	**Lesion size**	**Lesion type**	**Plaque stability**	**Incidence**	**Long(L) /Short(S) term**	**Advantages**	**Limitations**
**MOUSE**								
*ApoE*^−/−^ (35–39)	CD (8–10 w)(3–4 m)	n.i. 3,157 ± 437 μm^2^	n.i.Type II	Stable	Plaque 100%	S (4–5 w),L (3–4 m)	- Higher total cholesterol levels than *Ldlr*^−/−^ - Severe hypercholesterolemia - Diet hyper-responsive	- Only homozygous mice display the phenotype - *ApoE*^−/−^ impacts immune response - No xanthomatosis - Hypercholesterolemia is much more severe than the human phenotype - Occurring sudden deaths are unpredictable and differ greatly
	WTD (8–10 w)	9,200 ± 4,700 μm^2^	Type V	Stable	Plaque 100%	S (4–5 w)	- Severe hypercholesterolemia - Lesions are consistent, pronounced, and more widespread	
	WTD (8–48 w)	1,19,300 ± 17,800 μm^2^ 1,75,400 ± 9,800 μm^2^	Type VI	Unstable	Plaque 55.4% 78.8%	L (16 w)L (40 w)	- Provides a model for intervention studies for unstable plaques	
*ApoE*^−/−^*Fbn1C1039G*^+/−^ (40–44)	WTD (6–26 w) ♀ only	AAo 2,21,000 ± 35,000 μm^2^ DAo 17–49 × 10^3^ ± 15–18 × 10^3^ μm^2^ BCA 1,93,000 ± 29,000 μm^2^	Type V	Stable	Coronary plaques & MI (<75%)	S (10 w)	- Disturbed cerebral blood flow (<73% of cases) - Brain hypoxia in 64% of cases, indicating stroke - Mimics pathology of aged vessels - Spontaneous plaque rupture - Intraplaque neovascularization and hemorrhage	- Long-term WTD <70% sudden death in mice (increasing with longer WTD), whereas controls survived - Head tilt, disorientation, and motor disturbances (66% of cases) - Phenotypes vary strongly from study to study
	CD (6–26 w)	AAo 7,62,000 ± 51,000 μm^2^ BCA 2,25,000 ± 10,000 μm^2^	Type V	Stable	Plaque rupture, rarely	L (20 w)		
	WTD (6–26 w)	AAo 8,14,000 ± 60,000 μm^2^ BCA 4,56,000 ± 22,000 μm^2^	Type VI	Unstable	Plaque rupture AAo 70% BCA 50%			
ApoE*3-Leiden (45–48)	WTD (10–34 w) ♀ only	15,000 μm^2^	Type IV	Stable	n.i.	L (24 w)	- Diet inducible hyperlipidemia	- Reported poor breeding in homozygotes - Great variation of plaque progression (no lesions-severe lesions) - No spontaneous plaque rupture
	WTD (8–28 w)	Ø 5 × 10^4^ μm^2^	Type 0-V	Stable	Type 0, 30%;Type I–III, 65%;Type IV–V, 5%	L (19 w)		
	CD (8–24 w) ♀ vs. ♂	♀ Ø 1 × 10^4^ μm^2^ ♂ Ø 1,000 μm^2^	n.i.	Stable	n.i.	S-L (4–28 w)		
	WTD (8–24 w) ♀ vs. ♂	♀ Ø 12 × 10^4^ μm^2^ ♂ Ø 500 μm^2^	n.i.	Stable	n.i.			
ApoE*3-Leiden.CETP (46, 47)	WTD (8–28 w)	Ø 28 × 10^4^ μm^2^	Type I-V	Stable	Type I–III 30%; Type IV–V 70%	L (19 w)	- Lipoprotein cholesterol distribution is more human-like - VLDL cholesterol levels are very susceptible to dietary cholesterol levels - CETP is a pro-atherogenic factor in ApoE*3-Leiden mice - Diet inducible hyperlipidemia	- Decreased SR-BI-mediated cholesterol efflux - Gender-dependent phenotype - No spontaneous plaque rupture
	CD (8–24 w) ♀ vs. ♂	♀ Ø 1 × 10^5^ μm^2^ ♂ Ø 7,500 μm^2^	n.i.	Stable	n.i.	S/L (4–28 w)		
	WTD (8–24 w) ♀ vs. ♂	♀ Ø 18 × 10^4^ μm^2^ ♂ Ø 3,000 μm^2^	n.i.	Stable	n.i.			
Tandem stenosis in *ApoE*^−/−^ (3)	HFD (6–12 w); 150 μm, 450 μm TS (12–23 w)	n.i.	Type V	Stable	Plaque 100%; 14%	L (8–17 w)	- Mimics type VI atherosclerosis - Models intraplaque hemorrhage (IH)	- Surgery must be performed on each mouse
		n.i.	Type VI	Unstable	IH (50.6%, 0%) Rupture (32%, 0%)			
AVV-*PCSK9^*DY*^* (49)	HFD (0.75% chol.) (60–144 day)	C57BL/6J 350 ± 30 μm^2^ *ApoE*^−/−^ 710 ± 150 μm^2^	n.i.	Stable	n.i.	L (8 w)	- Stable PCSK9^DY^ mRNA expression - Persistent IDL/LDL hyperlipidemia - No long-term breeding needed to generate the model - Can be performed in different genetic backgrounds, but C57BL/6J seems to be most susceptible to PCSK9^DY^ - Synergistic effect of *ApoE*^−/−^ and PCSK9^DY^	- Lesion development requires a longer time than in *ApoE*^−/−^ - No spontaneous plaque rupture - Gender-dependent phenotype
		112 ± 27 μm^2^; n.i.	n.i.	Stable	n.i.	L (12 w) L (<1 year)		
Injected to *Tie2-rtTA/TRE-Gpr124* (50)	WTD; (n.i.)	7 × 10^3^ μm^2^	Type III	Stable	n.i.	L (16 w)	- Increased serumlevels of TC and LDL-C	
*Ldlr*^−/−^ (24, 36)	CD	n.i.	Type I–II	Stable	n.i.	S (2 w), L (<1 year)	- Elevated plasma cholesterol - Diet hyper-responsive - Elevated apoB-100, apoB-48, and apoE levels	- No Xanthomatosis - No spontaneous plaque rupture
	PD	n.i.	Type V	Stable	n.i.	S (2 w), L (6–8 m)	- Xanthomatosis - Greatly increased apoB-100, apoB-48 and apoE levels	
*ApoE*^−/−^*Ldlr*^−/−^ (51)	CD (16–28 w)	Ø 11.7 × 10^5^ μm^2^	Type IV	Stable	n.i.	L (10 w)	- LCHP could cause inflammation-driven unstable plaque rupture	- No spontaneous plaque rupture with WTD
	WTD (16–28 w)	Ø 15.2 × 10^5^ μm^2^	Type V	Stable	n.i.			
	LCHP (16–28 w)	Ø 20.2 × 10^5^ μm^2^	Type V	(Stable)	n.i.			
**Zebrafish**								
WT (28, 52–54)	HCD, adults; larvae (30 dpf)	100–500 μm (length)	Type II	Stable	Plaque 75%	S (5–14 day); L (<10 w)	- Hypercholesterolemia - Accelerated lipid oxidation - 2-week HCD leads to up to 70x increase of oxCE - Phenotype in adults and larvae	- Only mimics beginning stages of atherosclerosis
*apoc2*^−/−^ (28, 55)	ND; larvae (3–14 dpf)	n.i.	Type II	Stable	n.i.	S (11 day)	- Hypercholesterolemia - LCAT deficiency limits HDL cholesterol efflux capacity - Free cholesterol (FC)/CE ratio is increased as in LPL-def. patients	- Impacts ISV and SIV growth in early developmental stages, which is corrected 14 dpf - Only mimics beginning stages of atherosclerosis
*ldlr*^−/−^ (29)	No feeding (<5 dpf)	n.a.	n.a.	n.a.	n.a.	S (5 day)	- Hypercholesterolemia - Increased susceptibility to HCD	- Only mimics beginning stages of atherosclerosis
	ND, Larvae (4.5–9 dpf)	n.i.	Type II	Stable	n.i.	S (5 day)	- Moderate, sign. increase of vascular lipid deposits	
	HCD, Larvae (4.5–9 dpf)	n.i.	Type II	Stable	n.i.	S (5 day)	- Dramatic increase of vascular lipid deposits	

*AAo, ascending aorta; DAo, descending aorta; BCA, brachiocephalic arteries; ISV, intersegmental vessel; SIV, subintestinal vessel; CD, Chow diet; HCD, high cholesterol diet; LCHP, low-carbohydrate, high protein; ND, Normal diet; PD, Paigen diet; WTD, Western-type diet; n.a., not applicable; n.i., no information*.

The major apolipoprotein in chylomicrons, Apolipoprotein E (*ApoE*), can serve as a ligand for the LDL receptor (LDLR). Synthesis of *ApoE* occurs in the brain, liver, and other tissues in both humans and mice, although humans have high-density lipoprotein (HDL) subsets and *ApoE* isoforms that mice lack (56, 57). In addition to its effects on lipoprotein metabolism, *ApoE* has roles in macrophage biology, immune function, and adipose tissue biology (56, 58). Mutations in mouse *ApoE* result in human-like phenotypes, such as hyperlipoproteinemia type III, xanthomatosis, or dysbetalipoproteinemia (35). At the age of 3 months on a normal diet, *ApoE*-deficient mice show signs of type II fatty streaks (Figure 1). Type III diffusely intimal foam cell deposits in the aortic sinus can be observed at 5 months. Finally, at 8 months, the atherosclerotic plaques in *ApoE*^−/−^ mice seal the artery almost completely. The relatively early development of lesions and the anthropomorphous lipid metabolism are major advantages of the mouse as an animal model (35). Yet, lesion rupture is usually only observed under severe stress (30).

Along with LDL, *ApoE* is a structural component of all lipoproteins and a critical ligand for hepatic clearance of plasma lipoproteins, and it is mediated by LDLR (59). Hepatic clearance may explain why mice are naturally resistant to atherosclerosis (36). Deficiency of LDLR, more common in humans than non-functional *ApoE*, leads to reduced uptake and clearance of lipoproteins, resulting in the predominance of LDL as a cholesterol carrier and to familial hypercholesterolemia, which in turn increases the risk of CVDs (59). The human-like lipid metabolism of *Ldlr*^−/−^ mice enables the study of macrophages loaded with cholesterol creating xanthomas in the skin and subcutaneous tissue, as well as atherosclerotic lesions in arteries (36). In 2017 Emini Veseli et al. confirmed the plaque rupture in 20-week-old *ApoE*^−/−^*Fbn1C1039G*^+/−^ and *Il1r1*^−/−^*ApoE*^−/−^ mice, that mimicked human type VI atherosclerosis (17). Fragmentation of elastic fibers in the vessel wall, increases arterial stiffness and thereby leads to plaque rupture in 50% of the brachiocephalic arteries (40, 41). Inactivation of IL-1 signaling in atherosclerotic plaques decreased plaque collagen levels in *Il1r1*^−/−^*ApoE*^−/−^ mice, subsequently destabilizing the lesion (60); however, 50% of the mice die suddenly after receiving a HCD for 16–23 weeks (40). Lack of natural type VI atherosclerosis (i.e., lesion rupture; Figure 1), remained a detractor of the mouse model for many years. This changed not only with development of the *ApoE*^−/−^*Fbn1C1039G*^+/−^ and *Il1r1*^−/−^*ApoE*^−/−^ but also the tandem stenosis (TS) mouse model (3), which can mimic various stages of human atherosclerosis, including type VI lesion rupture (Figure 1). Chen et al. demonstrated that treatment with atorvastatin increased collagen content, and they concluded that plaque stability increased, although they observed no difference in total plaque area. Earlier studies showed that statins, combined with ezetimibe, could reduce lesion size by 17%, and had anti-inflammatory effects in mice, although there was no effect on serum oxLDL (61).

As an alternative approach Roche-Molina et al. (49) and Bjørklund et al. (62), established the AAV-*PCSK9*^*DY*^ model with adeno-associated virus (AAV) vectors serotype 8 resp. 9 carrying the human gain-of-function mutation Asp374Tyr in protein convertase subtilisin/kexin type 9 (*PCSK9*^*DY*^), that developed atherosclerosis and hyperlipidemia in the timespan of 84 day [Table 1; (49)]. Injection of *PCSK9*^*DY*^ into 30 day-old mice resulted in stable *PCSK9*^*DY*^ mRNA expression and persistent intermediate density lipoprotein (IDL)/LDL hyperlipidemia up to 1 year (49). Bjørklund et al. reported a lesion progression up to type V atherosclerosis (62). Since development of this approach it has been widely used, e.g., for highly efficient *PCSK9*-targeted genome editing via zinc-finger nuclease (ZFN) mRNAs, that were delivered to the liver by lipid nanoparticles (63); and to demonstrate the gender-dependent role of the EC-mineralocorticoid receptor (MR) for atherosclerosis and vascular inflammation, revealing the potential of EC-MR inhibitor therapy in male patients (64). Transexpression of human disease-causing genes in mice is a refined concept, that reduces time and number of animals required for the generation of mutants (65) and could provide a new effective and sustainable approach to study long-term atherosclerosis and hyperlipidemia (49) as well as to perform high-content experiments, in which it would reduce time and costs (62).

A particular disadvantage of genetically modified mouse models is that transport of cholesterol is mediated by HDL, rather than LDL (56), as lipid metabolism plays a crucial role in the development of lesions in the vascular wall. Recent publications report the use of very low density lipoprotein (VLDL) as the major plasma cholesterol carrier in *ApoE*^−/−^ mutants, as well as having atheroprotective properties (59). Another point to consider is passenger gene effects, which can arise due to various types of incestuous pairings (66). Passenger gene effects do not influence the fitness of an individual but can lead to subtle changes in the genetic background. This situation arises when a donor strain is bred to a recipient strain that carries the mutation of interest. The F1 generation is then bred again with the recipient strain, and repetition of this process for ten generations leads to a statistically 99.8% pure strain; however, 0.2% contamination from the donor strain remains. Considering crossover during meiosis, a small contamination can lead to additional variation in features such as cellular composition, calcification, and lesion size in the newly-developed strain (67, 68).

Very few animal model studies lead to new therapies and drugs, as mice and humans differ not only in size, maturation, and metabolism per gram of tissue (and in nutrient requirements), but also in telomere length and microbiome (31). For example, mice with human inflammation-associated diseases, such as lupus, psoriasis, and rheumatoid arthritis, develop more atherosclerotic lesions than patients with these conditions. Thus, promotion of vascular inflammation by atherosclerosis is more likely to result from general inflammation than from shared risk genes (69, 70). Among 25,000 compounds investigated in labs, only 25 will be tested in humans, of which five will come to market (71), corresponding to a success rate of 0.02% for individual compounds. One rare example of success is the development of PCSK9 monoclonal antibodies (mAbs) (alirocumab and evolocumab) in mice, which contributes to proven therapy for high LDL cholesterol (LDLc) levels and atherosclerosis in humans (56). While these mAbs are approved vaccines, their short *in vivo* half-time makes them expensive and thereby frequent administration is required (72). In recent years, two further PCSK9-based drugs were investigated in a clinical trial (ClinicalTrials.gov NCT02508896), the phase I of which was already completed in August 2017, although no results have yet been published. In ApoE^*^3-Leiden.CETP mice immunization using this peptide-based vaccine elicits antibodies against PCSK9, that reduced lesion size, especially decreased necrotic core-formation and macrophage inflammation and lowered plasma lipid levels through humoral response (72, 73). This approach promises to be more cost effective and might offer a long-term LDLc management (72).

### Zebrafish

The advantages of mice, such as easy care and breeding, and the ability to control the diet to induce atherosclerotic lesions, are shared by zebrafish (*Danio rerio*), making them another promising candidate animal model to study atherosclerosis.

The translucency of zebrafish larvae until about 30 days post-fertilization (dpf) permits observation of vascular development *in vivo* in real time (74, 75), which is augmented by the existence of transgenic lines such as *fli1:eGFP* and *lyz:DsRed2* (52, 76). The zebrafish is especially fecund, with females laying 200–300 eggs/day (77); however, excessive inbreeding leads to infertility (78), impeding the development of purer inbred lines. Although the vascular system of the zebrafish is different from that of mammals, its development and anatomy are similar (79).

During embryonic and first larval stages, zebrafish must rely on their innate immune system, since the adaptive immune system is not yet mature. Both adaptive and innate immune systems are highly conserved between zebrafish and mammals. The dominant leucocyte in zebrafish is the neutrophil, which responds to H_2_O_2_ gradients in injured tissues, while eosinophils provide an important host defense against parasites. Furthermore, macrophages are the key phagocytic cells that regulate cytokine-mediated immunity (80). In addition, zebrafish are likely to have dendritic and other antigen-presenting cells (81).

Zebrafish are ideal models for large-scale screens; the early larval stages are only 2–3 mm long, they develop rapidly, and their results are representative for mammals (82). Various genetic tools, such as MOs, targeting-induced local lesions in genes (TILLING), RNA caging, transgenic reporter lines expressing fluorescent marker proteins (i.e., the UAS/GAL4-system), ENU mutagenesis, the CRISPR/Cas9 system, and transcription activator-like effector nuclease (TALEN), facilitate evaluation of gene functions in zebrafish (83, 84). Like mammals, zebrafish process lipid throughout the intestine and hepatobiliary system (79, 83). Thus, they are often used to study vascular lipid accumulation and lipoprotein oxidation. In contrast to mammals, zebrafish cannot synthesize their own vitamin C and must obtain it from their diet; consequently, vitamin C levels can be manipulated to trigger oxidative stress [Table 2; (79)].

**Table 2:** Comparison of animal models for atherosclerosis.

	**Mouse**	**Zebrafish**		**Rat (57, 85–87)**	**Rabbit (88–92)**
		**Larvae**	**Adult**		
*ApoE*^−/−^	✓	**X**		✓	✓
*Ldlr*^−/−^	✓	✓		✓	✓
*Apoc2*^−/−^	✓	✓		**X**	**X**
*lcat*	✓	✓		✓	✓
*cetp*	**X**	✓		**X**	✓
Maturation	2 months	3 months		2–3 months	5–6 months
Progeny	5–8	200–300		Ø10	4–12
Transparency	**X**	✓	**X**	**X**	**X**
Housing	Group (6–10)	Swarm (4–10/L)		Group (2–4)	Group (2–8)
Dominating the lipoprotein profile	LDL	HDL		LDL	VLDL and LDL
Cholesterol transport	HDL	HDL		HDL	HDL
Vitamin C synthesis	✓	**X**		✓	✓
Favored source of energy	Carbohydrates	Lipid		Carbohydrates	Carbohydrates
Collection of blood samples	Non-lethal	None from individuals; lethal	Mostly lethal	Non-lethal	Non-lethal
Intima thickening	✓	**X**	✓	✓	✓
Lesions	Aorta and carotids	Caudal vein	Dorsal aorta	Aorta	Aorta and carotids
Lesion rupture	Rarely	**X**		**X**	Rarely
Highest atherosclerotic classification	Type V to type VI rupture	Type II fatty streak		Type III	Type V to type VI rupture
Oxygenated cholesteryl esters	In atherosclerotic lesions	In body liquids		In atherosclerotic lesions	In atherosclerotic lesions

In recent years zebrafish larvae, younger than 5 dpf, which are not considered animals until they start independent feeding according to the Directive 2010/63/EU of the European Parliament, have become a valuable animal model of drug discovery (93). Effects of administered compounds on vital developmental processes, such as vasculogenesis and organogenesis can be assessed in the whole organism during embryogenesis in a cost-effective and time-saving manner (93, 94). Today two approaches in terms of drug discovery are being pursued: Molecular target-based screens and high-throughput phenotypical screens. While performing target-based screens is fast and efficient, the risk that the compounds are eliminated downstream in the pipeline of preclinical and clinical trials is significantly higher than with phenotypic screens, as efficacy is lacking (95–97). Automated imaging systems and script-based evaluation tools improve the efficiency of phenotypic screens and thereby create a platform with enhanced data quality output, disease relevance, and reduced risk of elimination (95, 98).

Zebrafish animal models also have limitations. Collecting blood samples from juveniles from 40 to 45 dpf is possible, but lethal (52), and the larvae are so small that homogenates of several individuals are required for analysis. Another disadvantage of zebrafish is that poikilothermic vertebrates favor lipids as a source of energy, whereas carbohydrates are favored by homoeothermic mammals (52). Furthermore, mammals and fish differ not only in terms of favored energy source but also in their lipoprotein profile and LDL makeup. In zebrafish, their lipoprotein profile is dominated by HDL and their LDL contains more triglycerides and fewer cholesteryl esters (CEs) relative to human LDL (52). This is because zebrafish HDL functions as the cholesterol transporter, while in mammals it transports excess cholesterol from peripheral cells back to the liver, where it is recycled and excreted (99). Feeding a HCD to WT zebrafish causes their lipoprotein profiles to more closely resemble those of humans (74) and results in hypercholesterolemia, vascular lipid accumulation, and myeloid cell recruitment, among other symptoms (100). In both zebrafish and human, cholesteryl ester transfer proteins (CETPs) are involved in the transfer of CE from HDL to other lipoproteins (30, 101), which may explain why a HCD results in hypercholesterolemia in WT zebrafish.

All major classes of apolipoproteins (ApoA, ApoB, ApoC, and *ApoE*) are expressed in zebrafish and are highly similar to human apolipoproteins (79). As mentioned above, hypertriglyceridemia is an independent CVD risk factor caused by single mutations in the genes encoding lipoprotein lipase (LPL) or in those encoding LPL cofactors, such as APOC2 (28, 55). Liu et al. demonstrated that *apoc2*^−/−^ zebrafish exhibit properties of human patients, including dyslipidemia, specifically hypertriglyceridemia as early as 14 dpf, which resulted in type II atherosclerosis. To examine whether vascular lipid deposits accumulated in macrophages, they crossed *apoc2*^−/−^ with Tg (*mpeg1*-*eGFP*), that expresses eGFP in macrophages and emerged them in BODIPY to fluorescently stain neutral circulating lipoproteins and intracellular lipid droplets *in vivo*. They were able to show a co-localization of vascular lipid deposits with macrophages in *apoc2*^−/−^ *mpeg1*-*eGFP* 14 dpf as well as IK17 co-localization in selected areas of the larval fatty streaks, which is observed in early atherosclerotic lesions in human and mice (28, 100). In concordance other studies demonstrated a significant increase of LDL concentrations at vascular bifurcations (LDL concentration polarization) *in vivo* in larvae 52 hpf after fluorescent DiI-LDL-injection 48 hpf (5) and after feeding larvae fluorescence-labeled HCD short term (10 day) and long term (<10 weeks) (53).

Recently, Liu et al. reported the first *ldlr*^−/−^ zebrafish mutant, created using the CRISPR/Cas9 system, thus introducing a new model for atherosclerosis and hypercholesterolemia, that resembles the corresponding model in mice (24, 36). This mutant exhibits elevated susceptibility to HCDs and vascular lipid accumulation at 9 dpf, after 5 days of feeding a HCD (29).

Overall, studies conducted on zebrafish larvae may only be able to complement mammalian studies (102). Nevertheless, eight compounds discovered in zebrafish drug screens are being tested in clinical trials (93) and a promising approach showed that persistent expression of the human monoclonal antibody IK17 prevents vascular lipid accumulation and reduces existing lipid deposits in zebrafish (100). It is suspected that IK17 accumulates in vascular lesions and neutralizes oxidation-specific epitopes (74). Further, reduction in oxLDL, mediated by IK17, is predicted to decrease foam cell formation.

### Atherogenic Immune Responses in Mice and Zebrafish

Very few studies have characterized the whole immune response involved in atherosclerosis in zebrafish, hence our comparisons of them with mice and humans are also based on non-CVD focused studies (Table 3).

**Table 3:** Immune response factors in human atherosclerotic lesions.

	**Human**	**Mouse**	**Zebrafish**		**Human**	**Mouse**	**Zebrafish**
B-cells	✓	✓	✓	TLR7	✓	✓	✓
T-cells	✓	✓	✓	TLR9	✓	✓	✓
Granulocytes	✓	✓	✓	MyD88	✓	✓	✓
Dendritic cells	✓	✓	✓	MARCO	✓	✓	✓
Macrophages	✓	✓	✓	CD36	✓	✓	✓
E-selectin	✓	✓	✓	SRA-1	✓	✓	✓
P-selectin	✓	✓	✓	SRA-2	✓	✓	n. i.
VCAM-1	✓	✓	Vcam1a/b	SR-B1	✓	✓	✓
CCL2	✓	✓	✓	MCP-1	✓	✓	n. i.
CCL5	✓	✓	✓	IL-1α/β	✓	✓	✓
CXCL10	✓	✓	n. i.	IL-6	✓	✓	✓
CX3CL1	✓	✓	n. i.	IL-8	✓	✓	✓
TLR1	✓	✓	✓	IL-12	✓	✓	Il-12a/b
TLR2	✓	✓	✓	IL-17	✓	✓	Il-17a/f2
TLR3	✓	✓	(Tlr22)	TGF-β	✓	✓	✓
TLR4	✓	✓	Tlr4b.a/b	IFN-γ	✓	✓	Ifn-γ1-2
TLR5	✓	✓	Tlr5a/b				

*n. i., no information could be obtained for these factors. This table was created, using the information provided by the following sources: (8, 15, 80, 103–108), www.enseml.org*.

In the early stages of atherosclerosis, T_H_1-induced adhesion molecules, such as P-selectin and E-selectin, are highly conserved from zebrafish to mammals and have important roles in the inflammatory response (109). Mammalian chemokines, such as CXCL11, have homologs in zebrafish (CXC-64), although initial functional analyses revealed differences in their release from monocytes following stimulation by bacteria (103). The TLR family is conserved from insects to mammals; however, their signaling pathways can differ. Li et al. analyzed the PRRs and their corresponding homologs in mammals and zebrafish, and found that some TLRs have two counterparts in zebrafish, due to gene duplication events during evolution (104). They identified the TLR22 of zebrafish as a homolog of mammalian TLR3. Although mice lack TLR10, they have three additional TLRs: TLR11, TLR12, and TLR13 (110). TLR4 is required for efficient lipid uptake by macrophages in humans, mice, and zebrafish (52). Myeloid differentiation primary response 88 (MyD88) is a key pro-atherosclerotic adaptor protein in signaling cascades mediated by IL-1R and various TLRs (105).

The phylogenetically-conserved autoantigens, Hsp60 and LDL, stand out as important promoters of atherosclerosis. Hsp60 functions as a chaperone at the cell surface, folding freshly-synthesized proteins, and protecting them under stress conditions such as heat shock. In some cases, Hsp60 can trigger adaptive and innate immune responses *in vivo* (111). Studies of Hsps in mice lead to preclinical trials of DiaPep277, a vaccine, that had the potential to change Hsp-mediated immune regulation and was intended to treat type-I diabetes (112), but was terminated due to scientific misconduct (113).

In mice, immunization against oxLDL attenuates atherosclerosis. Phospholipids are ubiquitous molecules that form integral parts of cell membranes and lipoproteins such as LDL. SRs of macrophages, such as CD36, TLR2, and TLR4, take up oxidized phospholipids (oxPLs) and oxLDL, thereby promoting inflammation because they are recognized by the innate immune system. Knockout of SRs that recognize oxPLs leads to abatement of atherosclerosis in both models. oxPLs are found in all types of atherosclerotic lesions, indicating their contribution to atherogenesis (114). Accessory proteins found in humans are also present in zebrafish, indicating similarities in the activation and folding of zebrafish TLRs. The SR accessory protein, CD36, fine-tunes TLR assembly, especially in response to TLR2/6 ligands (106), and drives atherosclerosis through the aforementioned interaction with oxLDL (107, 115). It was shown, that while highly conserved, alternative splicing may not occur and differences in C-terminal amino acids of Cd36 may alter the function of the protein to being a co-receptor in contrast to mammalian CD36 (107).

## Discussion

In this review, we compared the most important aspects of mouse and zebrafish models for human atherosclerosis. In the last decade, models mimicking human atherogenesis, different diets, and tools for forward and reverse genetics were also developed, accelerating, and increasing the efficiency of research progress.

Currently, mice are the most commonly used atherosclerosis model because mutant strains are susceptible to dietary interventions, and plaques progress to type V lesions in the aorta and carotids (35). Nevertheless, the development of late-stage lesions takes months, even when mice are fed a HCD and rupture is usually only observed under severe stress (30). Since mouse immune responses and pathways are similar to those of humans, they are a good model for studying atherosclerosis-related inflammatory responses, whereas the HDL-based lipid metabolism and the lack of CETP in mice are clear disadvantages, that are only abolished in ApoE^*^3-Leiden.CETP (Table 1). Increased use of the relatively new *PCSK9*^*DY*^ mouse model negates the need to generate inbred strains. One recent example is the use of *PCSK9*^*DY*^-injections into mice expressing GPR124 under control of the Tie-2 promoter. Gong et al. found that GPR124, a receptor known to increase angiogenesis in the brain, influences the pathogenesis of atherosclerosis by activating nitrosative stress and NLRP3 inflammasome signaling (50). Nevertheless, while *PCSK9*^*DY*^ itself is suitable to create atherosclerosis models user errors and gender-dependent susceptibility to PCSK9^DY^ could lead to great variations of study outcomes between laboratories (64, 65).

Zebrafish have recently emerged as models for atherosclerosis and are also susceptible to diet, and they have the added advantage of their amenability to high-throughput screenings. The adaptive and innate immune systems are highly conserved between zebrafish and mammals. Use of transgenic zebrafish lines that can track macrophages and neutrophils *in vivo* has recently provided new insights into the early development of atherosclerosis. Chronological *in vivo* imaging over a 10-day period revealed, that neutrophils rather than macrophages may accumulate in the sub-endothelial space before LDL concentration polarization (53). These results illustrate the importance of appropriate *in vivo* models for studying the pathophysiology of complex diseases such as atherosclerosis.

Similarities with humans in lipid metabolism and expression of CETP, along with the development of the *ldlr*^−/−^ and *apoc2*^−/−^ mutant, demonstrate other advantages of this new model. Furthermore, the data from the *apoc2*^−/−^ mutant reported by Liu et al. suggests, that zebrafish suitably and even more favorably than mice, model human dyslipidemia, a major risk factor for atherosclerosis (28, 116, 117). While mutant mice can exhibit atheroprotective properties, which can bias experimental results, passenger gene effects may not affect atherogenic zebrafish models because WT animals are already affected by HCD, negating the for inbred strains.

With advancing technology, zebrafish could also be treated with a TS approach to test whether the phenotype of atherosclerotic plaques will also be enhanced in this model. Haemodynamic forces in the arterial wall of zebrafish can be measured using transgenic lines, BODIPY, imaging systems and analysis tools such as ZebraLab (5, 28). The recent creation of the *ldlr*^−/−^ zebrafish mutant will enhance the importance of zebrafish as an animal model for atherosclerosis. *ldlr*^−/−^ mutants on a regular diet develop moderate hypercholesterolemia, whereas even short-term consumption of a HCD results in hypercholesterolemia and aggravated accumulation of lipids in the vascular wall. Therefore, *ldlr*^−/−^ as well as *apoc2*^−/−^ zebrafish could enable high-content screens for novel therapeutics and drug repositioning for atherosclerosis. Research on zebrafish atherosclerosis is heavily leaning on the lipid metabolism as the lesions are very small, due to the models size and disease progression limitations. On one hand, this could be a drawback for the model depending on the hypothesis, but on the other hand its easy genetic manipulation and opacity offers an opportunity to study early atherosclerosis pathomechanisms using e.g., transgenic lines, BODIPY, or injections with fluorescent dyes, into the bloodstream of larvae 48 dpf.

Considering the very low costs associated with a model that can be used at just 9 dpf, even laboratories with low budgets could drive research on atherosclerosis forward using zebrafish. The development of *ApoE*^−/−^ zebrafish would create a new tool for atherosclerosis research that could be compared with mouse models. To date, there is no mouse model with consistent plaque instability, while the *ApoE*^−/−^ mouse mutant appears to be key to the development of a transgenic model that completely mirrors human atherosclerosis. Feeding *ApoE*^−/−^ mice a HCD is sufficient for them to exhibit complex and multi-layered lesions, but loss of ApoE makes this model much more complex, as it's an integral part of the immune response and lipid metabolism (118). Nevertheless, the mortality rate of this mouse line varies greatly for the same diet and length of the experiment (Table 1). While Van der Donckt et al. reported no dead animals, which is consistent with our own experience, Stöhr et al. published a 20% mortality and Johnson et al. a mortality rate of 70% after 10 months on WTD (37, 38, 40). Other reports of WTD for shorter periods show the same variability in mortality rates (42, 43). It is unclear whether this variation is a consequence of the *ApoE* KO or husbandry differences.

Studies of the atheroprotective characteristics of oxLDL antibodies, which were demonstrated in mice and rabbits, could also be performed in zebrafish, followed by immunization studies (53, 119). Furthermore, functional analysis of chemokines in growing plaques and regulation of their promoters in zebrafish could further drive atherosclerosis research.

The use of new imaging systems and tools, such as magnetic resonance imaging (MRI), Ultrasound biomicroscopy (UBM) and improved contrast enhancers for computerized tomography (CT) will provide more reliable information about plaque size and position *in vivo*. Currently gold nanoparticles (AuNP) are most commonly used as contrast agent for CTs, as they can attenuate X-rays, are rarely toxic for humans and can target specific tissues when combined with fluorescent dyes and surface molecules (120). Several toxicity studies in zebrafish embryos revealed size- and chemical-dependent biocompatibility and toxicity (121), resulting in small, under-pigmented eyes, neurological defects, and abnormal behavior activity (122–124). Studies in mice showed, that the use of AuNP, such as AuroVist™ can visualize macrophage infiltration in vascular bifurcations in mice *in vivo*. However, scans have to be performed pre- and post-contrast injection, which increases radiation time for each animal and thus may increase the mortality rate if an animal has to be examined several times within a study. Additionally animals must be fixed in place to reduce repositioning effects, that impact readout of the scans (125, 126). Taken together, a contrast agent for CT is needed that reduces the burden on animals to enable a time-lapse recording of plaque progression *in vivo* with reduced influence on the survival rate and pathogenesis for a reasonable price. Another imaging technique that has been used for decades in humans and mice is MRI. It enables differentiation of plaque components based on their biophysical and biochemical composition without radiation *in vivo* (127). In recent years new techniques have been developed, that not only increased the resolution, but enable MRI of adult zebrafish as well (128). Repeated MRIs with and without use of non-toxic, gadolinium-based contrast agents, were performed without side-effects in mice and zebrafish (128–131). Altogether, MRI is a well-established imaging technique, that provides in depth information for several animal models as well as for humans, but interpretation of the data still needs a lot of experience (127) and the images are static and cannot display hemodynamics. Another imaging technique, that allows to calculate blood flow based on Doppler blood velocity and vessel diameter as well as detection of atherosclerotic plaques is the multifrequency UBM combined with Doppler ultrasound (132). Readout quality from UBMs is frequently increasing, enabling reliable diagnosis of small animals with higher heart rates and smaller vessel sizes (133–136). Nevertheless, interpretation of the acquired data requires even more experience. To date, the best imaging techniques with the highest resolution, such as microCT and light-sheet microscopy, are still lethal or only applicable to one model (137–139). While *in vivo* imaging of mice can be achieved by adapting human imaging technologies and *vice versa*, imaging of zebrafish larvae can be achieved with simple microscopes. Automated microscopes enable high-throughput imaging of the small fish with high quality data output. Therefore, zebrafish are more commonly used for drug discovery. However, they also have limitations, that do not apply to murine models. Drug screening in zebrafish only succeeds if the compounds to be tested are predominantly water-soluble or are injected into the fish. Additionally, in-water dosing could yield unpredictable exposures (93). Furthermore, protocols to conduct high-content screens in zebrafish are very diverse, therefore results in different laboratories may vary. Taken together, zebrafish are a good model for primary drug screens, provided that all limitations have been considered. Moreover, primary screens in zebrafish reduce the number of mice needed for secondary drug screening.

Overall, mice will continue to be pioneers to study atherosclerosis pathogenesis until the majority of the mechanisms underlying atherosclerosis in zebrafish have been elucidated and mutant animals have been established. Zebrafish have clear advantages in studying the influence of lipid metabolism on atherosclerosis. Therefore, the model should be chosen according to the hypothesis and a combination of mouse and zebrafish experiments should be considered for in depth studies. Additionally, it is important to find an *in vivo* imaging method that meets the challenges of different models and at the same time creates a platform that delivers consistent, reliable, and above all comparable results, regardless of the model chosen.

## Author Contributions

ZA and VV contributed conception and design of the review. VV collected data, organized the database, and wrote the manuscript. All authors contributed to manuscript revision, read, and approved the submitted version.

## Conflict of Interest

The authors declare that the research was conducted in the absence of any commercial or financial relationships that could be construed as a potential conflict of interest.
